# Impact of *Naja nigricollis* Venom on the Production of Methaemoglobin

**DOI:** 10.3390/toxins10120539

**Published:** 2018-12-15

**Authors:** Harry F. Williams, Paul Hayter, Divyashree Ravishankar, Anthony Baines, Harry J. Layfield, Lorraine Croucher, Catherine Wark, Andrew B. Bicknell, Steven Trim, Sakthivel Vaiyapuri

**Affiliations:** 1 School of Pharmacy, University of Reading, Reading RG6 6UB, UK; h.f.williams@pgr.reading.ac.uk (H.F.W.); d.ravishankar@reading.ac.uk (D.R.); h.j.layfield@reading.ac.uk (H.J.L.); 2 Venomtech, Discovery Park, Sandwich, Kent CT13 9ND, UK; paul.hayter@talktalk.net; 3 School of Biosciences, University of Kent, Kent CT2 7NZ, UK; a.bains@venomtech.co.uk; 4 BMG LabTech, Buckinghamshire HP19 8JR, UK; lorraine.croucher@bmglabtech.com (L.C.); catherine.wark@bmglabtech.com (C.W.); 5 School of Biological Sciences, University of Reading, Reading RG6 6UB, UK; a.b.bicknell@reading.ac.uk

**Keywords:** Snakebite, venom, methaemoglobin, haemoglobin, neglected tropical disease, spitting cobra

## Abstract

Snakebite envenomation is an affliction currently estimated to be killing upwards of 100,000 people annually. Snakebite is associated with a diverse pathophysiology due to the magnitude of variation in venom composition that is observed worldwide. The haemolytic (i.e., lysis of red blood cells) actions of snake venoms are well documented, although the direct impact of venoms on haemoglobin is not fully understood. Here we report on the varied ability of a multitude of snake venoms to oxidise haemoglobin into methaemoglobin. Moreover, our results demonstrate that the venom of an elapid, the black necked spitting cobra, *Naja nigricollis,* oxidises oxyhaemoglobin (Fe^2+^) into methaemoglobin (Fe^3+^) in a time- and concentration-dependent manner that is unparalleled within the 47 viper and elapid venoms evaluated. The treatment of venom with a reducing agent, dithiothreitol (DTT) is observed to potentiate this effect at higher concentrations, and the use of denatured venom demonstrates that this effect is dependent upon the heat-sensitive proteinaceous elements of the venom. Together, our results suggest that *Naja nigricollis* venom appears to promote methaemoglobin production to a degree that is rare within the Elapidae family, and this activity appears to be independent of proteolytic activities of venom components on haemoglobin.

## 1. Introduction

Snakebite envenomation is considered to be a major neglected tropical disease. It is estimated to affect nearly five million people and result in approximately 100,000 deaths annually [1]. It is a crisis that predominantly impacts upon people living in rural areas of developing nations, and can induce major socio-economic ramifications [2,3]. Despite the significant impact on the health and well-being of humans, much remains unknown about the different toxic proteins and peptides that are the major components of snake venoms [4]. Venomous snakes have been classified into several families and sub families, but the vast majority of deaths occur as a result of bites from those found in Viperidae (Vipers) and Elapidae (Elapids). These two families are very different; vipers are typically ambush predators that inject a venom composed mainly of large proteins that predominantly affect haemostasis, while elapids are generally more active hunters, with venoms containing fewer of these haemotoxic components but instead dominated by neurotoxic and cytotoxic proteins and peptides [5]. Thus elapids are less commonly associated with haemostatic disturbances.

A range of biological activities have been found to be induced by snake venom components, with many of these proteins targeting the blood, haemostasis and cardiovascular system [6,7]. Serine proteases and metalloproteases are the most abundant proteins found in viper venoms and they exhibit various haemotoxic effects. Metalloproteases are able to affect the basement membrane of blood vessels via collagenolytic activity [8], and several other venom components inhibit or activate platelets by different mechanisms [9,10,11]. As well as interacting with platelets and blood vessels, many venom proteins affect the blood coagulation cascade via coagulatory or anti-coagulatory effects [12]. An array of different proteins capable of causing haemolysis also exist within snake venoms, for example, the enzymatic phospholipase A2 (PLA2) which directly cleave the phospholipid bilayer, and the hydrophobic three-finger toxins, which can bind to the cell membrane to promote lysis [13]. Indeed, haemolysis can take place to such a degree that haemolytic anaemia has developed in some recorded cases of snakebites [14]. Haemolysis is a well-known effect of several snake venoms, although their direct effects on red blood cells (RBCs) and their components are not fully understood, especially given the oxidative stress typically observed as a secondary effect of certain snake bites [15,16,17].

In addition to natural intra- or extravascular haemolysis (approximately 10% of RBCs will lyse as a consequence of wear and tear) and direct lysis by venom components, haemolysis also takes place as a result of the venom-induced local tissue damage which produces reactive oxygen (ROS) and nitrogen species that contribute to oxidative damage and further lysis of cells [16]. The release of haemoglobin (Hb) into the plasma following haemolysis triggers inflammation and oxidative stress as well as impairing endothelial function. Excessive plasma Hb levels is called haemoglobinemia and it can induce extreme nitric oxide consumption and clinical sequelae including haemolysis-associated vasculopathies and endothelial dysfunction [18]. Free plasma Hb is bound rapidly by haptoglobin in order to inhibit its oxidative activity, and this complex is rapidly removed by the mononuclear phagocytic system, particularly the spleen [19]. After plasma haptoglobin is saturated, the excessive Hb is filtered out by the kidneys. Frequently, during envenomation these organs are already under pressure due to decreased renal blood flow as a result of ischaemia, thrombotic microangiopathy and rhabdomyolysis. These in turn cause myoglobin deposits in renal tubules [4] and together lead to acute kidney injury, which is a main systemic complication and is frequently the cause of death in viper bites [20].

RBCs are essential for the transportation of oxygen to the tissues and delivery of CO_2_ (or dissolved HCO_3_^−^) to the lungs; they are entirely dependent upon the oxygen transport protein, Hb. This protein relies on a prosthetic ferrous (Fe^2+^) haem group, the ferric oxidation of which renders the protein (termed as methaemoglobin (MetHb)) unable to transport oxygen (Fe^3+^), a process which has already been observed in specific snake venoms [21]. This process takes place at a very low rate naturally, and MetHb is maintained at <1% by rapid reduction to the ferrous form, a process catalysed by the enzyme methaemoglobin reductase [22]. Through this reaction, a superoxide free radical is generated but is quickly dismutated via the enzyme superoxide dismutase forming H_2_O_2_ and O_2_ [23]. Methaemoglobinemia occurs when the levels of MetHb occur above 2% and serious neurological and cardiovascular symptoms can develop as a result of hypoxia with levels of approximately 15% and above [24].

Venom-induced oxidative stress appears to be an effect of envenomation that anti-snake venom (ASV) (the only available treatment for snakebite envenoming) fails to ameliorate [25], and may actually exacerbate [21]. These effects are reduced by the co-administration of melatonin, an anti-oxidant agent, along with ASV, and there is evidence to suggest a reduction in methaemoglobinemia as a result [16,21]. However, the scale of oxidative stress and other effects of venom components on the functions of RBCs and Hb are yet to be studied in detail. Here, we report the screening of a large number of snake venoms and specifically characterise the effect of the venom of *Naja nigricollis* (*N. nigricollis*) to determine its impact on the production of MetHb.

## 2. Results

### 2.1. A Range of Snake Venoms Induce the Oxidation of Hb

**Figure 1:**
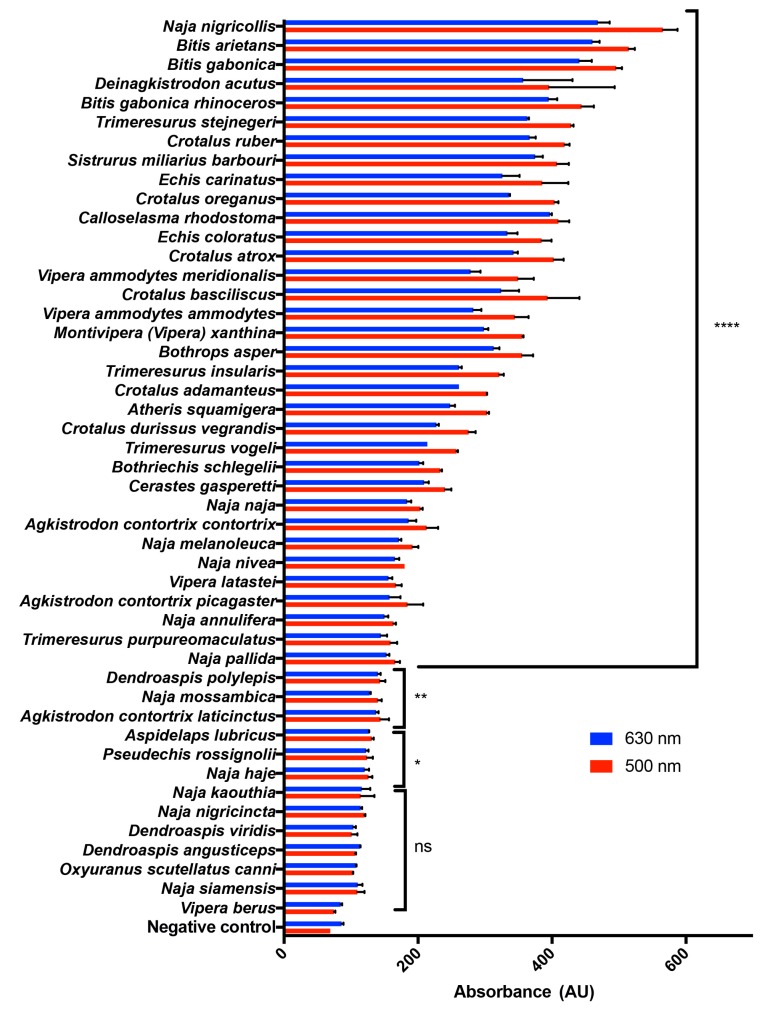
Impact of T-VDA^CV^ venom array on MetHb production. Whole snake venoms (0.2 mg/mL) from the T-VDA^CV^ array (Venomtech Limited, UK) targeting the cardiovascular system were dispensed into 96-well micro titre plates, mixed with an equal volume of ovine Hb, and incubated at 37 °C for 16 h. The absorbance was measured at 500 nm and 630 nm (i.e., the peaks typically observed for MetHb) by spectrofluorimetry (FLUOstar Optima, BMG Labtech) at 16 h. Data represent mean ± SEM (*n* = 3), and the *p* values (all the values were compared to the negative control [PBS]) were calculated by One-way ANOVA followed by post-hoc Tukey’s test using GraphPad Prism (* *p* < 0.05, ** *p* < 0.01, and **** *p* < 0.0001).

The ability of venoms to oxidise Hb into MetHb has been reported within the family of Viperidae [15,16,17,26]. Here, we screened 47 different viper (30) and elapid (17) venoms from the targeted venom discovery array for the cardiovascular system (T-VDA^CV^) in order to determine the impact of these on MetHb production. The venoms (0.2 mg/mL) were mixed with an equal volume of ovine Hb (the supernatant of lysed ovine RBCs) and incubated for 16 h prior to measuring the absorbance at 500 nm and 630 nm (i.e., absorbance maxima for MetHb). An overnight period of 16 h was used in the experiments in order to determine the maximum impact of venom on MetHb production. The data suggest that Hb oxidising activity is common among several viper venoms but this is a rare phenomenon for an elapid snake venom (Figure 1). Notably, the large increase in absorbance observed in many viper venoms was only evident in the venom of one elapid, *N. nigricollis*. As this is a unique feature observed in *N. nigricollis* venom, we have analysed this effect in greater detail.

### 2.2. Naja Nigricollis Venom Is Significantly Increasing MetHb Production

**Figure 2:**
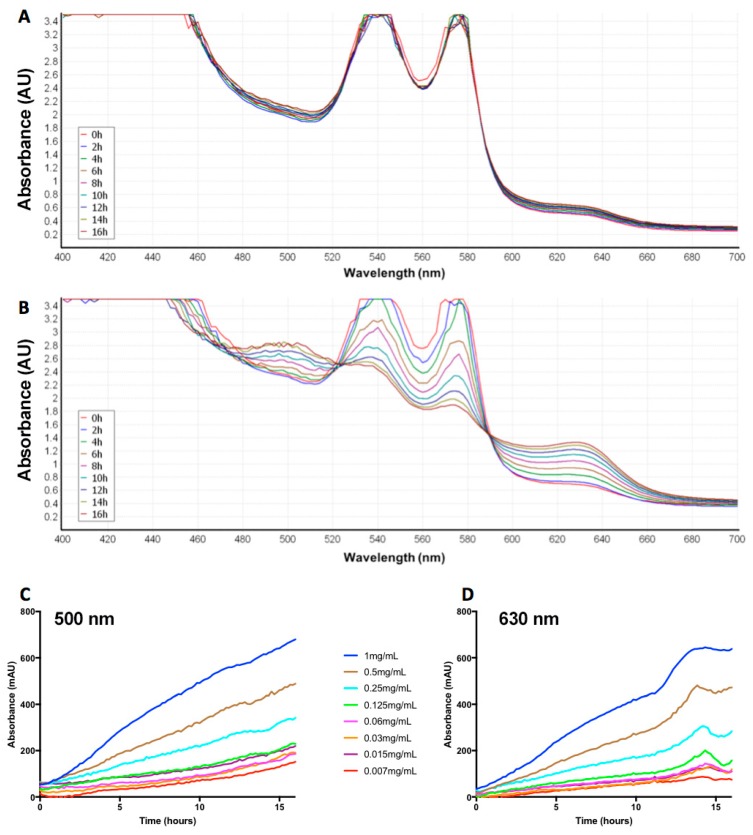
Time- and concentration-dependent changes in absorbance for MetHb over 16 h. Ovine Hb was incubated with a control (PBS) (**A**) or *N. nigricollis* venom (10 mg/mL to achieve maximum MetHb production) (**B**) at 37 °C in a plate reader (PHERAStar, BMG Labtech). The absorbance between 400 nm and 700 nm was measured every 2 h. The peaks typical of oxygenated Hb are at 540 nm and 570 nm, and those of MetHb are at 500 nm and 630 nm. Furthermore, the *N. nigricollis* venom was serially diluted from 1 mg/mL to 0.007 mg/mL and mixed with an equal volume of ovine Hb. It was then incubated for 16 h at 37 °C in a plate reader (FLUOStar Optima, BMG Labtech) and the absorbance was measured every 2 h at 500 nm (**C**) and 630 nm (**D**). The traces shown are representative of three separate experiments, and they were selected to clearly demonstrate the changes in absorbance for Hb and MetHb over a period of 16 h.

To determine the effect of *N. nigricollis* venom on MetHb production, additional experiments were performed using ovine Hb. *N. nigricollis* venom (10 mg/mL was used to achieve a maximum response) was incubated with ovine Hb and the level of absorbance for Hb and MetHb was measured every 2 h for 16 h. The venom displayed a time-dependent decrease in absorbance at 540 nm and 570 nm (i.e., peaks observed for Hb) with a corresponding time-dependent increase in absorbance at 500 nm and 630 nm (i.e., peaks typical of MetHb) (Figure 2A,B). Similarly, the increasing concentrations of *N. nigricollis* venom suggest that this venom-induced impact on MetHb production is also concentration-dependent (Figure 2C,D). Therefore, an optimal concentration of 0.2 mg/mL venom was used in other experiments in this study. Together these results suggest that the *N. nigricollis* venom is promoting the oxidation of oxyhaemoglobin to MetHb in a time- and concentration-dependent manner.

### 2.3. Heat-Denaturation of N. nigricollis Venom Reduces Its Effect on MetHb Production

**Figure 3:**
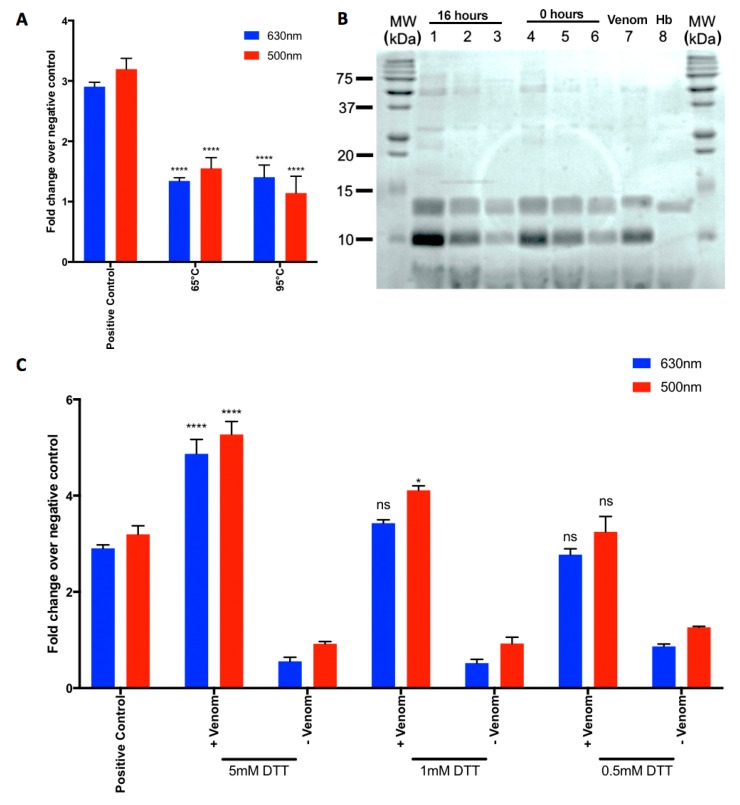
Effect of heat, proteolytic activity of venom and a reducing agent (DTT) on MetHb production. (**A**) *N. nigricollis* venom was heat treated at 65 °C or 95 °C for 10 min prior to cooling the venom to room temperature and mixing with ovine Hb. The level of absorbance at 500 nm and 630 nm was measured after 16 h. (**B**) SDS-PAGE (Coomassie stained) gel showing the protein profile of Hb before (as a control) and after incubation with different concentrations of *N. nigricollis* venom for 16 h. Lanes, MW—molecular weight marker, Lane 1 and 4: Venom (12 µg) + haemoglobin (3 µg). Lane 2 and 5: Venom (6 µg) + haemoglobin (3 µg). Lane 3 and 6: Venom (3 µg) + haemoglobin (3 µg). Lanes 1-3; after 16 h incubation and lanes 4–6; as controls at 0 h. Lane 7: *N. nigricollis* venom (3 µg) alone and Lane 8: Hb (3 µg) alone. The image shown is representative of three separate experiments. (**C**) *N. nigricollis* venom was treated with various concentrations (0.5 mM, 1 mM or 5 mM) of reducing agent, DTT for 10 min prior to mixing with ovine Hb and incubating for another 16 h. The level of absorbance was measured as shown above. Data represent mean ± SEM (*n* = 3). The *p* values shown were as calculated by two-way ANOVA followed by post-hoc Tukey’s test using GraphPad Prism 7 (* *p* < 0.05, ** *p* < 0.01, *** *p* < 0.001 and **** *p* < 0.0001). The heated (**A**) or DTT (**C**)-treated samples were all compared with the respective positive controls (untreated venom), and the negative control used was PBS.

In order to determine whether the ability of *N. nigricollis* venom on MetHb production is dependent on proteins, this venom was heat-denatured prior to using in the assay. The venom was incubated at two different temperatures, 65 °C and 95 °C for 10 min and then cooled to room temperature prior to use in the assay with ovine Hb. Notably, the heat-denatured venom exhibited significantly reduced MetHb production at 16 h (Figure 3A). This data suggest that MetHb production may be mediated through proteins that are present in the venoms and they are sensitive to heat denaturation.

### 2.4. N. nigricollis Venom Does Not Exert Direct Proteolytic Activity on Hb 

To determine whether the MetHb production occurs due to proteolytic actions of *N. nigricollis* venom on Hb, different concentrations of the venom were incubated with 3 µg Hb overnight along with controls, and these samples were analysed by SDS-PAGE. As shown in Figure 3B, the venom does not have any direct proteolytic activity on Hb as the bands for this protein are intact and unaffected by increasing concentrations of venom over 16 h compared to the controls. These data suggest that *N. nigricollis* venom-induced effects on MetHb production are not dependent on direct proteolytic actions of this venom on Hb, although we cannot rule out the possibilities of the effects of this venom on other components that regulate MetHb production.

### 2.5. A Reducing Agent Increases the N. nigricollis Venom-Induced MetHb Production

To assess the impact of a reducing agent on the *N. nigricollis* venom-induced production of MetHb, 1,4-dithiothreitol (DTT) was used in the assay. The venom was pre-treated with different concentrations of DTT [we chose a range of concentrations (low to high) including 0.5 mM, 1 mM and 5 mM in order to analyse the maximum impact of this reducing agent on venom-induced MetHb production] and then incubated with ovine Hb. The presence of DTT failed to prevent the oxidation of Hb, and indeed increased the production of MetHb at the highest concentration (5 mM), although at lower concentrations this increase was not significant (Figure 3C). These data suggest that venom-induced MetHb production is not diminished by reducing agents such as DTT.

## 3. Discussion

The proteins found within the snake venoms have a multitude of pharmacological actions including direct effects on cells. These include cell lysis or blockade of their functions via binding to a range of receptors and ion channels. They also affect the blood coagulation cascade that is required for effective haemostasis and the cholinergic disruptions to nerve transmission [27,28]. Moreover, a number of snake venoms have been reported to induce direct haemolysis and lead to ischemia [29,30]. In this study, we have investigated the effects of a selection of snake venoms on the oxidation of Hb. Most of the snake venoms tested caused at least a minor increase of absorbance at the selected wavelength maxima for MetHb, although the elapids’ absorption generally increased to a much lesser extent than the vipers. Despite this, compared to the other viper and elapid venoms, the largest shift observed was for the venom of an elapid, *N. nigricollis*.

**Figure 4:**
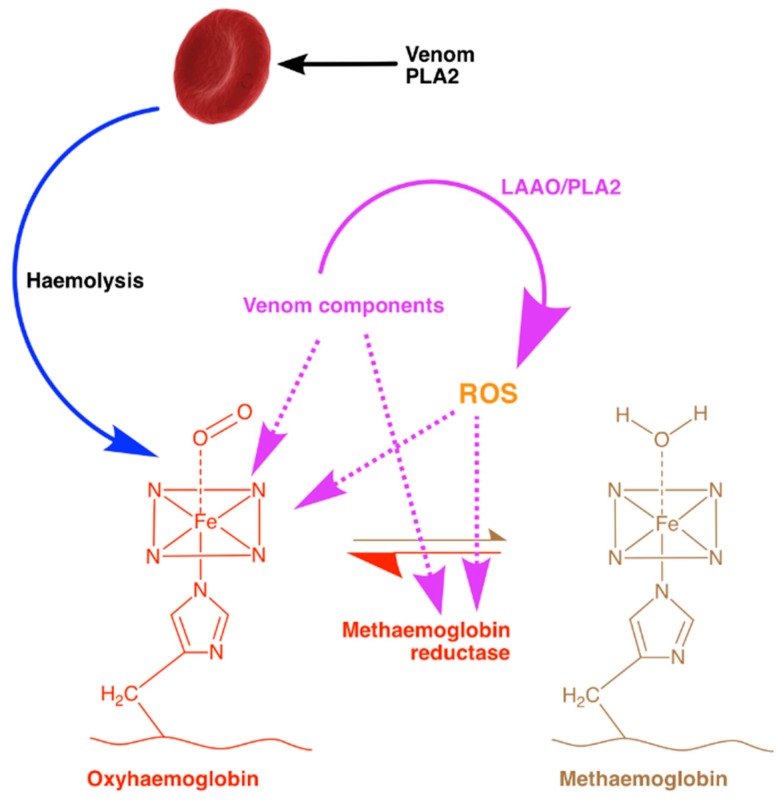
**A schematic diagram hypothesising the impact of venom on MetHb production**. Haemolysis (the lysing of RBCs) by phospholipase A2 (PLA2) and other venom components is a common effect of snakebite envenoming. In a healthy human, the oxidation of Hb to MetHb is constantly combated by an enzyme, MetHb reductase. After certain venomous snakebites, this equilibrium is shifted in favour of MetHb, a toxic species that is also known to be pro-inflammatory. This shift may be a result of the ROS, which is generated as a metabolite of l-amino acid deamination [via l-amino acid oxidases (LAAO)] or lipid peroxidation (via PLA2) present in various venoms. The potential mechanisms for venom-induced changes in MetHb production are shown in the figure as dotted magenta lines.

In a previous study [21], *Naja naja* (Indian Cobra) venom was found to have a greater ability to produce MetHb than two other Indian vipers (*Echis carinatus* and *Daboia russelii*). Those results are inconsistent with the findings from this study where many of the viper venoms including *Echis carinatus* had more pronounced effects than *Naja naja* venom. Although the reasons for these discrepancies are not entirely clear, they have used human whole blood in their experiments, whereas we have used ovine Hb. Therefore, their experiments may relate more to the ability of venoms to lyse RBCs (as Hb would be much more accessible after lysis) than to directly oxidise Hb. We can postulate that single or multiple venom components may act either directly on Hb or potentially on MetHb reductase to prevent the equilibrium found under healthy natural conditions. A schematic diagram of our working hypothetical model of the impact of venom components on the production of MetHb is shown in Figure 4.

A previous study using *Crotalus molossus nigrescens* venom reported that high concentrations of this venom cause some level of proteolytic degradation to Hb [26]. The same study postulated that the venom’s conversion of Hb to MetHb is due to the oxidative stress induced by H_2_O_2_ which is produced by venom LAAO [26]. It may be possible that following this, H_2_O_2_ then reacts with the ferrous Hb producing further reactive species via the Fenton reaction [31]. However, *N. nigricollis* is a snake with potently cytotoxic venom that lacks LAAOs and is composed largely of three-finger toxins (73.3%) and PLA2 (21.9%) [32]. These proteins typically bind to cell membrane components or directly affect the phospholipid bilayer which leads to cell lysis, although they have not been documented interacting directly with Hb. The lack of LAAO and the abundance of PLA2 in *N. nigricollis* venom supports the theory that the phospholipase activity could be indirectly causing this effect as a result of its oxidative product, arachidonic acid. However, members of the genus *Agkistrodon* screened for their Hb oxidising ability (Figure 1) were found to have little or no effects on Hb despite having a high percentage (31–46%) of PLA2 [33], more than half of which are enzymatic [34]. There is a very small percentage of group III metalloproteases (2.4%) and cysteine-rich secretory proteins (CRiSPs) (0.2%) found in *N. nigricollis* venom [32], components that are also found in viper venoms and are more typically associated with this oxidative effect. The metalloproteases cause multiple pathologies which contribute to oxidative stress including haemorrhage, thereby inducing spontaneous haemolysis, liberating Hb and free iron. Metalloproteases are also able to induce necrosis [35], releasing myoglobin which has implications on the redox balance. After the degradation of the extra-cellular matrix by metalloproteases, damage-associated molecular patterns (DAMPs) are likely to be released, and these can induce further oxidative stress and inflammation [25]. Given the lack of proteases in *N. nigricollis* venom, the highly cytotoxic PLA2 found in this venom is more likely to be the primary cause of this oxidation. Hydrolysis of the sn-2 acyl bond of plasma membrane glycerophospholipids by PLA_2_ releases free fatty acids such as arachidonic acid as well as lysophospholipids and lysophosphatidylcholine, and potentially toxic, ROS [25]. The increase in polyunsaturated fatty acids has also been postulated to cause an increase in lipid peroxidation following administration of a viper (*Echis carinatus*) venom, and it may be possible that similar peroxidation takes place as a result of *N. nigricollis* venom.

The time- (Figure 2A,B) and concentration- (Figure 2C,D) dependent shift in the absorbance of Hb/MetHb by *N. nigricollis* venom demonstrates that the components of this venom are able to promote the oxidation of Hb. The increase in MetHb production observed over 16 h and at higher venom concentrations suggests that this effect may increase over time in snakebite victims, and is dependent on the volume of venom injected by the snake. The increase in MetHb production observed with the venom that was treated with the highest concentration of DTT suggests that this effect is actually improved by the addition of a redox reagent. However, at lower concentrations of DTT, this increase was not observed. We originally hypothesised that the addition of a redox reagent such as DTT would prevent the production of ROS, and consequently MetHb by taking the place of antioxidants such as glutathione found naturally in the plasma [36]. The thiol groups of glutathione act as reducing agents in order to prevent the damage to cellular components by ROS. Therefore, the surprising increase in MetHb production with the highest concentration of DTT could be due to the reduction of the tetrameric structure of Hb and consequently, its enhanced oxidation. The decrease in activity seen with heat treatment of venom provides evidence to suggest that this effect may be due to the proteins present in the venom. The incubation of venom with purified bovine Hb and further analysis suggest that this effect is not a result of proteolytic degradation of the Hb. However, the results of a previous study using a protease-rich viper venom show that high venom concentrations can lead to proteolytic degradation of Hb. The SDS-PAGE presented here suffers from large protein bands (~16 kDa) in the crude venom overlapping with the Hb monomer (16.1 kDa), although it is clear that Hb is still present. Further investigation using non-reducing or native protein electrophoresis is required to ascertain if there is any degree of proteolysis with *N. nigricollis* venom on Hb and to further scrutinise the mechanism of action of this venom on MetHb production.

Venom-induced neurological and coagulation pathologies have long been the two major areas of interest for scientists to investigate snakebite pathophysiology due to their prominence as the most immediately life-threatening aspects of a snakebite. However, the morbidity is gaining interest recently as estimates of as many as 15,000 people annually suffering amputations as a result of snakebites in sub-Saharan Africa alone [37]. Hence, the disability adjusted life years (DALYs) and loss to any snakebite-afflicted region’s work force are of serious importance. ASV is currently the only therapy for snakebite envenoming [4], and while it is effective in saving lives when administered appropriately, it frequently fails to address some of the long-term damage induced by snake venom components. This includes myotoxicity and skeletal muscle damage [38,39] as well as oxidative stress [25], which puts undue pressure on the body that is already suffering from an array of potentially fatal venom-induced health consequences. Following haemolysis caused either directly by the lysis of RBCs or as a result of ROS produced by venom components, Hb levels are elevated in the blood. As a result, this reduces the oxygen-carrying ability of the blood and decreases its ability to meet the oxygen demand of vital organs. Furthermore, the elevated levels of Hb cause a reduction in free-haptoglobin levels and can overwhelm the mononuclear phagocytic system, the spleen and eventually the kidneys, which may lead to acute kidney injury [40]. The additional complication of conversion to MetHb could potentially allow this toxic Hb species to build up and affect the efficiency of the systems in place to reduce free Hb. These complications to the blood would then undoubtedly have further effects on haemostasis and exacerbate existing ischaemic areas, contributing in turn to tissue damage, necrosis and long-term damage from snakebites.

In conclusion, we have demonstrated that a wide variety of venoms possess Hb-modifying activity among a range of viper and elapid snakes. From the venoms tested, we have observed a significantly higher activity for *N. nigricollis* venom for the production of MetHb. This effect is likely to be a result of proteins present in the venom, although further fractionation and characterisation is required to identify the exact proteins causing these changes. Moreover, most of the experiments performed in this study used ovine Hb due to availability. However, future experiments will be performed using human RBCs and Hb. The clinical significance of venoms on MetHb production and its contribution to envenomation will also be determined in the future. The work highlighting co-administration of melatonin with ASV is worth pursuing further [21], and the use of antioxidants in vivo could potentially be developed further to ascertain whether the oxidative stress can be reduced using flavonoids and their derivatives [41]. Although it may be a less important effect of snakebite envenomation than the immediately life-threatening elements, the reduction of oxidative stress on internal organs and tissues following a snakebite is likely to improve recovery and potentially reduce some of the long-term damage and morbidity as a result.

## 4. Materials and Methods

### 4.1. Materials

The cardiovascular targeted-venom discovery array (Catalogue code: T-VDA^CV^) of whole snake venoms was from Venomtech Limited (Sandwich, UK) and ovine blood was obtained from Envigo (UK) Ltd. (Oxon, UK). Clear 96- and 384-well plates were purchased from Greiner (Gloucestershire, UK), and DTT, purified bovine Hb, and all other chemicals were from Sigma Aldrich (Dorset, UK)

### 4.2. Ovine RBC Lysis and Hb Production

Ovine blood was lysed by the addition of two volumes of ultrapure water followed by centrifugation (5000× g), and the clear supernatant was collected and used in further assays as ovine Hb. Although this supernatant is likely to have other components from RBCs, the presence of a prominent Hb band was confirmed by SDS-PAGE prior to using this as a Hb source in further functional assays.

### 4.3. Screening of Venoms for MetHb Producing Activity

The whole snake venoms from the T-VDA^CV^ array were diluted in 1× PBS (Fisher Scientific, Loughborough, UK) (0.2 mg/mL) and mixed with an equal volume of ovine Hb (diluted 1:2 in 1× PBS) with a final assay volume of 100 µL in a 96-well micro titre plate. These samples were then incubated at 37 °C and the absorbance at 500 nm and 630 nm was measured at 16 h by spectrofluorimetry (FLUOstar Optima, BMG Labtech, Aylesbury, UK).

### 4.4. Time- and Concentration-Dependent Effect of N. nigricollis Venom on MetHb Production

Ovine Hb diluted (1:2) in 1× PBS was incubated with *N. nigricollis* venom (at 10 mg/mL or a range of concentrations between 1 mg/mL and 0.007 mg/mL) or PBS (a negative control) in a 384-well plate. Plates were incubated at 37 °C in a plate reader (PHERAStar, BMG Labtech, Aylesbury, UK) and the absorbance spectrum between 400 nm and 700 nm was measured at 2-h intervals over 16 h. After this time, maximum Hb oxidisation appeared to be reached, and therefore, this time point was chosen for other experiments in this study. Moreover, to determine the impact of heat and a reducing agent, venom was heat-treated (65 °C or 95 °C in a dry bath) or treated with a reducing agent, DTT (0.5 mM, 1 mM or 5 mM) prior to using them in assays. The temperatures (65 °C or 95 °C) were determined based on our previous experience in denaturing venom proteins and using them in functional assays. DTT was specifically selected as this is a less toxic and volatile compared to β-mercaptoethanol; moreover, DTT prevents the reoxidisation of disulfide bonds in proteins.

### 4.5. Sodium Dodecyl Sulfate-Polyacrylamide (SDS-PAGE) Gel Electrophoresis

The SDS-PAGE analysis was performed according to standard procedures as described previously [42]. The samples were taken before and after incubating a range of concentrations of venom with purified bovine Hb (Sigma Aldrich, Dorset, UK) at 37 °C for 16 h. Samples taken prior to 16-h incubation were used as controls to compare the impact of venom on Hb before and after the incubation. These samples were mixed with reducing sample treatment buffer (Bio-Rad, Watford, UK) and run on a pre-made (Bio-Rad, Watford, UK) 10% SDS-PAGE gel for 40 min at 150 constant volts. Gels were then stained with Coomassie brilliant blue (Bio-Rad, Watford, UK) for approximately 1 h on a rocker, before destaining overnight (10% acetic acid/10% methanol/80% H_2_O).

### 4.6. Statistical Analysis

All statistical analyses were performed using GraphPad Prism 7. *p*-Values were calculated using one-way or two-way ANOVA followed by Tukey’s post-hoc multiple comparisons test.
